# What happens to the brain during pregnancy?

**DOI:** 10.1172/JCI183888

**Published:** 2024-07-15

**Authors:** Andrea M. Maxwell

**Affiliations:** University of Minnesota, Minneapolis, Minnesota, USA.

In the true spirit of probably any MD/PhD student, the second I saw that second pink line emerge on my pregnancy test on an early Saturday morning, I started looking for data. In addition to the classic “new mom” questions (Prenatal genetic testing? Choline? Doula?), I fished out my medical school embryology textbook and provided my husband with unsolicited, daily updates on our exact stage of fetal development. I cracked open *Williams Obstetrics* to review perinatal clinical care. I even read up on placental physiology. For the most part, I found what I was looking for. Information regarding my fetus’s growth was ample. *Williams* had everything I wanted (and didn’t want) to know about childbirth mechanics. I learned all about the fetoplacental unit. But I couldn’t find, really, any information about my body above the neck.

What was happening to my brain? Why, suddenly, did I have to reread sentences multiple times before understanding them? Why did I start waking up with 3 AM “brain zoomies,” racing, intrusive thoughts that never occurred during the daytime? In my second trimester, I struggled to associate familiar names with faces. When I mentioned that I might have “pregnancy brain” to my husband, he responded, “Well . . . I didn’t want to be the first one to say it.” Apparently, my lapses had not gone unnoticed.

I dove into the neuroscience literature, intent on understanding the hippocampal brain circuitry changes that may underlie these memory problems, how brain network connectivity is modulated by progesterone levels, and, generally, the pattern of perinatal synaptic plasticity. As a neuroscience PhD student, I thought I was well equipped to do so. Instead, I found how little literature actually exists. With some exceptions that have been published within the past few years (1–5), very few neuroscientific studies have examined the human pregnant brain. In her book, *Mother Brain,* Chelsea Conaboy details the neuroscience of pregnancy, which is interwoven with her own experiences as a new mother (6). A frequent review of the book from the lay audience is *Where is the neuroscience*? And indeed, where is it? Despite Conaboy’s comprehensive review of the science, the community thirsts for more information. *What happens to the brain during pregnancy?*

Until relatively recently, examination of the live human brain was difficult. Functional magnetic resonance imaging (fMRI), however, in combination with improved computational methods that allow for individual (3) and population-level (7) analyses of brain changes, puts the study of the pregnant brain within reach. As of 2019, almost 85% of female individuals in the United States had a biological child before the age of 49 (8), and literature suggests that pregnancy may be a sensitive period of neurodevelopment akin to puberty, with long-lasting effects (7, 9). It is simply unacceptable to allow such a physiologically salient change in brain health remain so understudied.

Moreover, understanding the normal development of the pregnant brain is essential to understanding when that development goes awry. Suicide and opioid overdose, two heartbreaking outcomes of brain-based medical conditions, together are a leading cause of pregnancy-related deaths in the United States (10, 11). We need to identify neural markers of resilience and vulnerability across pregnancy if we want to develop neuroscientifically informed treatments for our pregnant patients. Indeed, as Molly Dickens, a stress physiologist and women’s mental health scholar, writes in her blog, *The Maternal Stress Project*, our understanding of even the basic physiology of cortisol, the quintessential “stress hormone,” across pregnancy is far from well understood (12), not to mention cortisol’s effect on perinatal brain pathophysiology. How are we to understand even more nuanced questions without these mechanistic data? My thesis work examines how stress and social support dynamically interact with brain structure and function in alcohol misuse. I hope to extend this work to study perinatal addiction in the future; however, our current knowledge of how each of these constructs present during pregnancy, even outside of addiction, leaves a lot to be desired.

I shouldn’t have been surprised by the lack of information. Pregnant populations have been systematically excluded from research (13), frequently under the auspices of protecting the fetuses; yet, too often, this exclusion occurs at the expense of the pregnant patient. We are more than just vessels for the developing baby; pregnant people deserve rigorous investigation into the mechanisms that define this momentous physiological and psychological change in our brain health. Fortunately, there is growing momentum in the field. The academic journal *Nature Neuroscience* recently highlighted an fMRI paper studying pregnancy brain changes on its front cover, public conversations about perinatal mental health are increasing, and the White House recently signed an Executive Order carving out federal funds for women’s health research (14). I hope that, if the baby kicking inside of me as I write this essay one day becomes a parent, he’ll get to do so in a world better equipped with the information and tools needed to support its pregnant community.

## Version 1 07/15/2024

Electronic publication
